# An EMI-Based Clustering for Structural Health Monitoring of NSM FRP Strengthening Systems

**DOI:** 10.3390/s19173775

**Published:** 2019-08-31

**Authors:** Ricardo Perera, Lluis Torres, Antonio Ruiz, Cristina Barris, Marta Baena

**Affiliations:** 1 Department of Mechanical Engineering, Technical University of Madrid, c/ José Gutiérrez Abascal, 2, 28006 Madrid, Spain; 2 Analysis and Advanced Materials for Structural Design (AMADE), Polytechnic School, University of Girona, Campus Montilivi, 17003 Girona, Spain; 3 Department of Geological and Mining Engineering, Technical University of Madrid, c/ Ríos Rosas, 21, 28003 Madrid, Spain

**Keywords:** Structural health monitoring, PZT sensors, electro-mechanical impedance, hierarchical clustering, k-means clustering, NSM-FRP strengthening

## Abstract

The use of fiber-reinforced polymers (FRP) in civil construction applications with the near-surface mounted (NSM) method has gained considerable popularity worldwide and can produce confident strengthening and repairing systems for existing concrete structures. By using this technique, the FRP reinforcement is installed into slits cut into the concrete cover using cement mortar or epoxy as bonding materials, yielding an attractive method to strengthen concrete structures as an advantageous alternative to the external bonding of FRP sheets. However, in addition to the two conventional failure modes of concrete beams, sudden and brittle debonding failures are still likely to happen. Due to this, a damage identification technology able to identify anomalies at early stages is needed. In this work, some relevant cluster-based methods and their adaptation to electromechanical impedance (EMI)-based damage detection in NSM-FRP strengthened structures are developed and validated with experimental tests. The performance of the proposed clustering approaches and their evaluation in comparison with the experimental observations have shown a strong potential of these techniques as damage identification methodology in an especially complex problem such as NSM-FRP strengthened concrete structures.

## 1. Introduction

In the last few decades, strong research efforts have been devoted to the continuous structural condition assessment for the civil engineering infrastructure [1]. Structural health monitoring (SHM) traditionally refers to the process of implementing monitoring systems to measure structural responses in real-time and to identify anomalies and/or damage at early stages [2].

By controlling and analyzing damage-sensitive features, extracted from raw experimental data on a monitored structure, it is possible to detect structural anomalies which may be due to damage. 

Within the field of strengthening of reinforced concrete (RC) structures with fiber-reinforced polymer (FRP) composite materials, some previous studies have been devoted to the identification of damage at their earliest stages on externally bonded (EB) FRP reinforcement. For this purpose, different non-destructive testing (NDT) methodologies such as infrared thermography [3,4] and ultrasonic testing [5,6] have been proposed. Most of these techniques require of a priori knowledge, at least approximate, of the possible damage locations and, additionally, the part of the structure to be inspected should be easily accessible; these disadvantages make difficult their application to large and complex structures for which the inspection might become dangerous and time consuming. To avoid this, a more feasible alternative, the electromechanical impedance (EMI) technique based on the use of PZT (lead zirconate titanate) transducers, has also been applied for FRP external strengthening [7,8,9,10]. PZTs can act as both actuators and sensors and yield an active sensing technique able to detect internal damage at low cost from the measurement of electrical impedances. In addition, as PZT transducers work exciting the structure in a high frequency range, their area of influence will be very local and close to the sensor and will not be affected by the vibrations coming from the outside environment such as vehicles and wind. Therefore, the EMI technique will be very useful to assess the health of areas nearby to each sensor. Furthermore, it might be easily implemented in a non-expensive way for continuous and on-line monitoring of large structures within a wireless system [11]. 

Until now, to the knowledge of the authors, no previous SHM work has been experimentally focused on the near surface mounted (NSM) technique using FRP. In the NSM-FRP methodology, the FRP reinforcement is installed into slits cut into the concrete cover using cement mortar or epoxy as bonding materials. Its use has several advantages over the EB FRP technique, such as protection, improved bond, better aesthetics, and surface preparation; due to this it has become an attractive method as an effective alternative for strengthening RC structures. 

De Lorenzis and Teng [12], and more recently Zhang et al. [13], provided a detailed and critical review of the current status and future research needs related to this technique. One important open issue concerning this methodology is with respect to the failure modes; the uncertainties in this area contribute to increase the misunderstanding of its long-term performance and decrease the confidence of design engineers in exploiting the full potential of FRP composite materials. Therefore, the implementation of a nondestructive periodic inspection methodology able to identify damage at the early stage might be very useful to mitigate these uncertainties.

A conventional global modal analysis, such as performed in [14] on reinforced-concrete (RC) beams strengthened with NSM-FRP rods subjected to different levels of damage, showed, as expected, a drop in frequency values due to damage. However, a local identification of the damaged areas would require to work in a higher frequency range than that used in the modal analysis. In this sense, the use of EMI method might provide information unable to be obtained with a conventional vibration analysis. When the local properties of the structure to be inspected change (due to damage, temperature, etc.), the impedance signatures captured with PZTs would also change and, therefore, might be used for damage identification. Traditionally, in most civil applications, the damage detection process is carried out using physics-based methods and parametric approaches. However, these methods require time and a high level of expertise and might not be practical when a large amount of information, as that obtained from PZT transducers, must be processed during a continuous monitoring. This is even more remarkable in a problem such as NSM strengthening where the behavior and the failure modes are dependent on a lot of variables and the impedance-based detection would require work with very refined and expensive numerical models able to simulate the high frequency structural behavior. Although some attempt has been made in the past for EB FRP reinforcement using this approach, the limitations are clear [15]. More frequently, different statistical metrics, such as the root mean square deviation (RMSD), the correlation coefficient (CC), or the covariance (Cov), have been used to characterize damage from changes of the impedance signatures [16,17]. However, the use of a single type of metric for EMI based identification has some limitations regarding damage location and severity. To avoid these problems, the use of simple methods able to appropriately identify in a direct and accurate way unusual patterns or outliers representative of damage from measured data and without requiring any input parameter is needed. These anomalies or outliers might be indicative of damage when applied to a structural health monitoring problem. With this purpose, nonparametric machine learning approaches are an interesting alternative to detect hidden patterns from monitored data and, therefore, to address damage assessment. 

Different machine learning approaches have been successfully applied for damage detection in the past [18,19]. Density-based approaches are based on the classification of the data according to their similarities using distance metrics, such as Euclidean or others; normal data points occur around a dense neighborhood and abnormalities are far away. Points representative of a normal condition are assumed to belong to a unique cluster or group. As an extension of these approaches, clustering is an interesting technique within the domain of unsupervised learning which can become very useful for anomaly detection [20,21,22,23]. By using a clustering approach, data points that are similar tend to be grouped in different subsets in such a way that the normal condition of a structure is characterized as clusters and the damage detection strategy is based on an outlier detection approach. 

Different alternatives exist to perform the clustering. In this work, some relevant cluster-based methods and their adaptation to EMI-based damage detection in NSM-FRP-strengthened structures SHM are developed. The proposed methodology is validated on datasets extracted from two RC beams strengthened with NSM-FRP experimentally tested under increasing loading until failure. 

The paper is organized as follows: In Section 2, a review of the EMI method for structural damage identification is introduced. The clustering approaches used in this work are presented in Section 3. The description of the experimental tests and the impedance datasets obtained from them are carried out in Section 4. Section 4 also describes the results obtained with the proposed approaches. Finally, in the last section, conclusions are presented.

## 2. Electromechanical Impedance Method

The electromechanical impedance technique is based on the self-sensing property of piezoelectric material which allows exciting a host structure using a PZT transducer attached to it and the acquisition of the response using the same PZT transducer. As defined in the 1-D theoretical model introduced by Liang et al. [24], the electrical admittance Y(ω) (inverse of impedance) of the PZT, computed from the excitation voltage V_i_ and the output current I_0_ of the PZT transducers, is directly related to the mechanical impedance of the structure Z_s_(ω) and, therefore, to the growth of structural damage, as follows:

(1) $$Y\left(\omega \right)=\frac{I_{0}}{V_{i}}=G\left(\omega \right)+\mathrm{jB}\left(\omega \right)=j\omega \frac{\mathrm{wl}}{h}\left({\bar{\varepsilon }}_{33}^{T}-\frac{Z_{s}\left(\omega \right)}{Z_{s}\left(\omega \right)+Z_{a}\left(\omega \right)}d_{3x}^{2}{\;\hat{Y}}_{\mathrm{xx}}^{E}\right)$$

where ω is the input frequency and w, l and h denote the width, length and thickness of the PZT patch, respectively; $d_{3x}^{2}$ and ${\hat{Y}}_{\mathrm{xx}}^{E}$ are the piezoelectric coupling constant and complex Young’s modulus of the PZT, respectively, and ${\bar{\varepsilon }}_{33}^{T}={\;\varepsilon }_{33}^{T}\left(1-\delta j\right)$ is the complex dielectric constant of a PZT patch under constant stress, where δ is the dielectric loss factor. Equation (1) shows also that the admittance signature is related to the mechanical impedance, Z_a_(ω), of the PZT patch although, usually, material properties of the PZT are assumed to be constant.

Additionally, PZT transducers excite the host structure within a high frequency range which makes EMI technique very attractive for local damage detection because of its sensitivity to even small damage [25,26,27,28,29,30,31,32,33]. However, it should be considered that ambient variations will also alter the impedance signal. This might make EMI method susceptible to false alarms which are no provided by mechanical damage and effects such as the temperature should be taken into account for structural health monitoring, especially in detecting low damage levels. In fact, the dielectric constant ${\bar{\varepsilon }}_{33}^{T}$ in Equation (1) is temperature-sensitive although it affects to the imaginary part of the impedance only. Because of it, the real part of the impedance signature has been reported to be more sensitive to mechanical damage than the imaginary part and is, therefore, more frequently used for damage identification purposes.

Different statistical metrics, such as the root mean square deviation (RMSD), the correlation coefficient (CC), the covariance (Cov), or the mean absolute percentage deviation (MAPD), have been used in the past to detect variations of the impedance signatures [34]. These variations can be identified with changes in the properties of the structure due to damage, variations in temperature, etc. These indices can exhibit different behaviors facing the changes of the impedance. Thus, for instance, the RMSD index is more sensitive to variations in the amplitude of the electrical impedance signatures while, on the other hand, the CC index is more sensitive to changes affecting to the shape of the signatures, such as frequency shifts. Although different studies have compared the performance of these indices as damage indicators, RMSD is the most frequently used in concrete structures.

## 3. Clustering Approach

### 3.1. Fundamentals

As commented previously, the use of the EMI method in combination with PZT transducers can become very interesting in detecting incipient damage prior to more catastrophic deterioration in NSM-strengthened RC structures due to their high frequency and localization capabilities. The use of a single type of metric (RMSD, CC, etc.) for EMI-based damage detection has some limitations which affect to the identification of damage location and severity. For instance, small damage at close range to the PZT transducer can present the same results as larger damage further away. Furthermore, evaluating quantitatively the damage degree can be very difficult using this approach. To solve some of these deficiencies, in this work, we propose the use of a novel non-model-based clustering method able to correlate the location and type of PZT transducers with their performance as well as with the level of damage of the structure to be analyzed. Basically, clustering is an unsupervised learning algorithm able to organize objects into groups according to their similarity or some recognized pattern.

The proposed clustering approach in this work is mainly based on two hypotheses. Firstly, on one hand, sensors located close to each other and under the same conditions are assumed to respond similarly to the same excitation if they are of the same type. According to this, the damage detection capability will be dependent on the type of sensor and on how the sensors are integrated with the host structure since PZT transducers can be directly attached to the surface of the structure or internally embedded. 

Based on the previous hypothesis, nearby sensors of the same type should have similar behavior and, therefore, should be grouped into the same cluster. Any measurement identified as an outlier might be interpreted as an anomaly or symptom of damage. 

Secondly, by using the same previous reasoning, measurements captured from the same sensor at different stages of the inspected structure are assumed to show a similar behavior except if some anomaly has occurred. If the environmental variability on the system is controlled, the anomalies should mainly be associated to the existence of mechanical damage.

According to the previous assumptions, potential anomalies can be identified by grouping clusters of similar sensors or, alternatively, clusters of similar stages for the same sensor. We think that combining both assumptions within a clustering framework, a procedure might be implemented able to increase the potential of correctly identifying and locating structural damage.

Different alternatives exist to perform the clustering. In this work, we will use two, an agglomerative or hierarchical algorithm and a k-means algorithm.

### 3.2. Hierarchical Clustering

By using a hierarchical clustering approach, each one of the different observations is initially treated as a single cluster. Subsequently, using an iterative procedure, nearby observations are merged into a new bigger cluster; then, successively, new nearby points are added to the nearest group and so on. In this sense, the algorithm uses a bottom-up or agglomerative strategy. Alternatively, a top-down or divisive approach might be followed, i.e., all observations start in one cluster and, recursively, new splits are formed as one moves down the hierarchy. The different levels of grouping can be visualized using a dendrogram, which is a sort of connectivity plot giving the big picture of the level of similarity between adjacent clusters. By cutting the dendrogram at different heights we can decide about how many clusters we want. Although this procedure can become computationally expensive, it produces very intuitive results which help to figure out which clusters combination makes more sense.

In an agglomerative methodology, the algorithm is as follows:

1. Assign each item or observation to a cluster. i.e., if we have N observations, we will have initially N clusters. In this way, distances between observations (similarity) will agree with distances between clusters.
2. Find the closest pair of clusters and merge them into a single cluster. The number of clusters will be reduced in one unity.
3. Compute the new distances between the new cluster built in step b) and the old clusters to get a new similarity framework.
4. Repeat steps b) and c) until grouping all observations into one single cluster or until reaching the number of desired clusters.

Step a) and reconfiguration of clusters in steps b) and c) require the use of a measure of dissimilarity between sets of observations; for it, a suitable distance metric and a linkage criterion must be defined. 

Different distance metrics can be applied. By choosing one or another the shape of the clusters will be affected. Usually, the cophenetic correlation coefficient, CCC, is used to define the best clustering method according to the distance measures. This coefficient is defined as the linear correlation between the dissimilarities, d_st_, between each pair of observations, s and t, and their corresponding cophenetic distances $d_{\mathrm{st}}^{\mathrm{coph}}$, which represent the intergroup dissimilarity at which the observations s and t first merged together in the same cluster:

(2) $$\mathrm{CCC}=\frac{\sum_{s<t}\left(d_{\mathrm{st}}-\bar{d}\right)\left(d_{\mathrm{st}}^{\mathrm{coph}}-{\bar{d}}^{\mathrm{coph}}\right)}{\sqrt{\sum_{s<t}{\left(d_{\mathrm{st}}-\bar{d}\right)}^{2}\sum_{s<t}{\left(d_{\mathrm{st}}^{\mathrm{coph}}-{\bar{d}}^{\mathrm{coph}}\right)}^{2}}}$$

In some way, this coefficient indicates the fidelity with which the clustering represents the distance matrix. In this work, good values have been obtained with the Manhattan or city block distance and, therefore, it has adopted in the study:

(3) $$d_{\mathrm{st}}=\overset{n}{\underset{j=1}{\sum }}\left|{\tilde{x}}_{\mathrm{sj}}-{\tilde{x}}_{\mathrm{tj}}\right|$$

According to Equation (3), considering n dimensions by observation, the Manhattan distance between two points or observations, s and t, is computed adding the distances corresponding to each dimension. In our study, for each loading stage, each observation is associated to one specific sensor.

Once the distances between observations belonging to the different clusters have been computed, the linkage criterion allows defining the dissimilarity between clusters from the pairwise distances of observations belonging to them. Different alternatives are possible. In this way, single-linkage and complete-linkage consider the distance between two clusters from the shortest and greatest distance from any point of one cluster to any point of the other cluster, respectively. On the other hand, if an average-linkage clustering is used, the distance between clusters is computed from the average distance between points of both clusters. This last approach has been used in this study.

### 3.3. K-Means Clustering

K-means is a widely used clustering algorithm in which the user decides a priori the number of clusters (k) to create. With this approach, those items or observations which fall outside of these groups might potentially be classified as anomalies. Unlike hierarchical clustering algorithm, only one parameter, k, is used in k-means.

The characteristic algorithm of this procedure is as follows:

1. Initialization of k centroids. This initialization might be performed randomly.
2. Allocation of all the observations to their closest centroid.
3. Updating of each centroid by computing the mean of all the items assigned to it.
4. Reallocation of the observations according to the updating of step c).

Steps c) and d) are repeated iteratively until reaching convergence, which means that no new reallocations are carried out in all the observations.

Additionally, for this clustering algorithm, once the grouping of the different sensors has been initially defined for a baseline condition, representing usually the healthy condition of the structure, a damage indicator can be assigned to each sensor and observation for subsequent stages using the centroids of the clusters as previously stated. For it, for any test observation vector, **s_i_**, associated to one sensor, the Euclidean distance to the centroid of the cluster to which the sensor belongs is calculated as:

(4) $$D_{k}\left(s_{i}\right)=\left(s_{i}-\mu_{k}\right)\Sigma_{k}^{-1}\left(s_{i}-\mu_{k}\right)$$

In this expression, the mean vector, **µ_k_**, and covariance matrix, **Σ_k_**, are computed for each cluster k from the values of the characteristic features of the members belonging to the cluster in a baseline stage, which might agree with the healthy structure or not depending on the purpose of the study.

Once Equation (4) is applied for each cluster, the damage indicator or index D for each observation is defined as the smallest value computed for all the clusters:

(5) $$D\left(s_{i}\right)=\min\left[D_{k}\left(s_{i}\right)\right]$$

Equation (5) is applied to each sensor and stage of the structure for which some impedance measurements have been performed. 

One important aspect to be addressed is about the choice of the clustering algorithm since significant differences occur among them. Either of them should be chosen depending on the requirements of the problem. In this work, we have used both agglomerative hierarchical and k-means algorithms, in a combined way, with the purpose of extracting the maximum amount of information possible of the impedance measurements given by the PZT transducers.

### 3.4. Feature Extraction

Before the application of any clustering algorithm, some meaningful attribute extracted from the impedance data captured by each PZT transducer should be extracted to perform the grouping process. The selected attribute should be especially sensitive to damage but not to operational and environmental conditions.

The collected data for each sensor and load case include five frequency sweeps providing five raw impedance signals. An average of the five signals has been performed and the real part of the average impedance signal was used as feature for the hierarchical clustering algorithms since it gives more information and is more sensitive to damage than the imaginary part.

For the application of k-means clustering, a meaningful feature extracted from the real part of the measured impedance spectrum (Re(Z(ω)) has been used for the grouping. In particular, moments of the measured impedance responses have been chosen as the feature in the k-means approach:

(6) ∫ω1ω2ωnRe(Z(ω))dω

These moments depend on the entire frequency range, (ω_1_, ω_2_), of the signal and, therefore, capture the information of the entire spectrum. To choose the order n of the moment, we have to take into account that the higher the order the higher the weight given to the higher frequencies. As we are interested in the local damage identification, high-frequency components are more suitable and, therefore, a moment with order 3–4 can provide useful information of the spectrum.

## 4. Case Studies and Results

**Figure 1:**
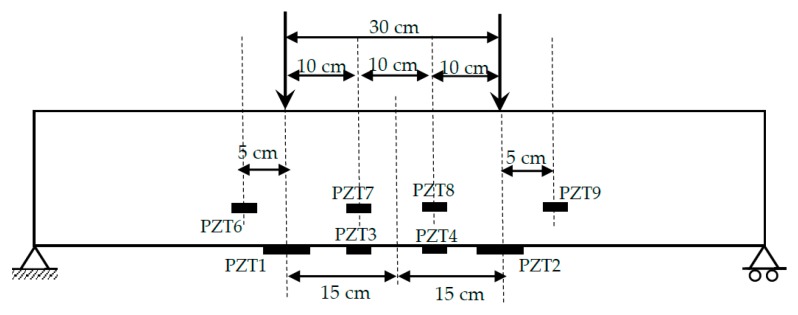
Test setup of specimen A.

**Figure 2:**
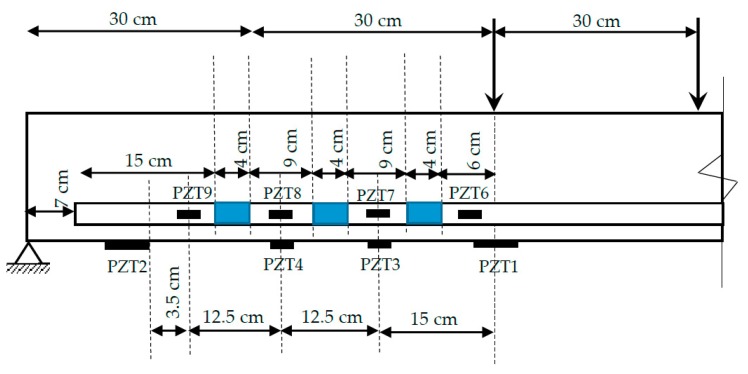
Test setup of specimen B.

An experimental study was carried out on two NSM-FRP specimens. One of the specimens (specimen A) (Figure 1) was expected to fail under bending by the fracture of the NSM bar at the intermediate section while, in the second specimen (specimen B), an initial debonded area at the FRP–concrete interface on the left side of the specimen, indicated in blue color in Figure 2, was induced before applying any load on it with the purpose of favoring the failure by debonding of the FRP reinforcement. Figure 2 shows only the portion of the beam between the left support and the applied load to represent the instrumented region in a more detailed way. However, in spite of this, specimen B failed by FRP bar rupture, which demonstrated the strong adherence properties introduced by NSM-FRP technique. The geometric dimensions and the reinforcement layout for both specimens are illustrated in Figure 1 and Figure 2, respectively. The material properties were assigned as follows: (a) for concrete, the elastic moduli and the compressive strength were taken to be E = 26 GPa and f_c_ = 30 MPa; (b) for steel reinforcement, the elastic moduli and elastic limit were taken to be E = 210 GPa and f_y_ = 510 MPa; (c) for the reinforcement, one CFRP bar with E = 170 GPa and tensile strength of 2500 MPa was inserted into the concrete cover.

**Figure 3:**
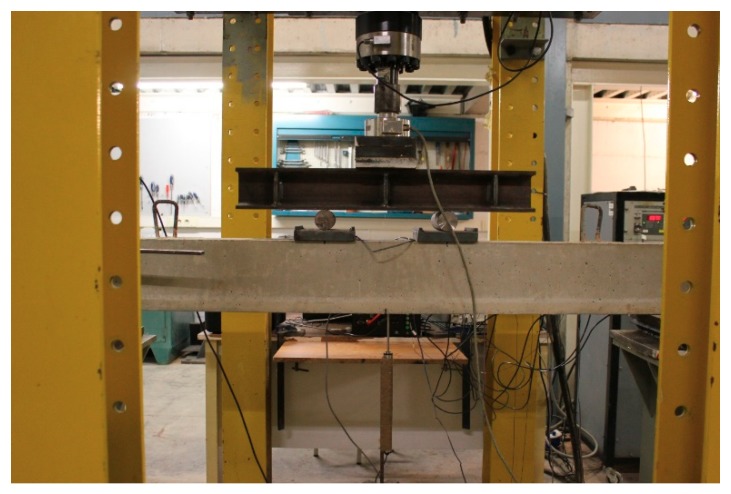
Experimental setup.

In the test program performed, the two strengthened beams were subjected to a series of four-point increasing static load tests with the purpose of gradually introducing deterioration into the specimens (Figure 3).

**Figure 4:**
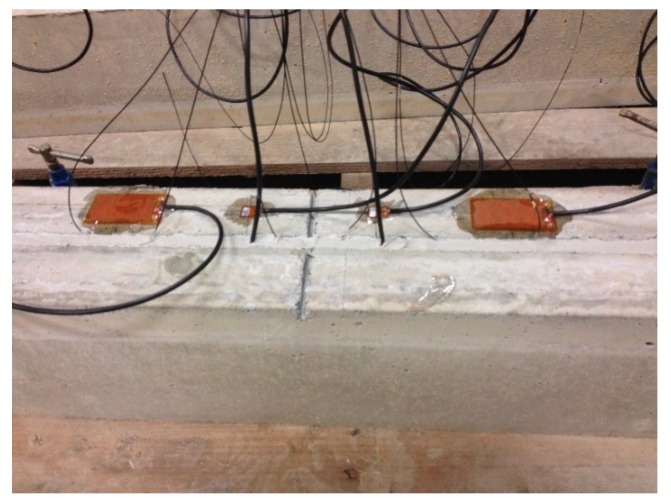
PZT-instrumented NSM-FRP specimen.

In order to verify the applicability of the proposed methodology, impedance measurements were captured from an array of eight PZT transducers once the beam has been unloaded after each static test and before loading it again. Two different transducer types and technologies of integration into the test specimens were applied. Two P-876.A12 DuraAct patch transducers of dimensions 61 mm × 35 mm × 0.5 mm (PZT1 and PZT 2 in Figure 1 and Figure 2) and six P-876.SP1 transducers of dimensions 16 mm × 13 mm × 0.5 mm (PZT3 to PZT9 in Figure 1 and Figure 2), both made by PI Ceramic (Lederhose, Germany), were used [35]. P-876.A12 transducers were bonded directly to the external face of concrete beam. Additionally, from the six smaller transducers, two were attached to the tangible surface while the other four were embedded into the structure by bonding them directly on the FRP bars/laminates. Figure 4 shows the PZT instrumented NSM-FRP specimen.

**Figure 5:**
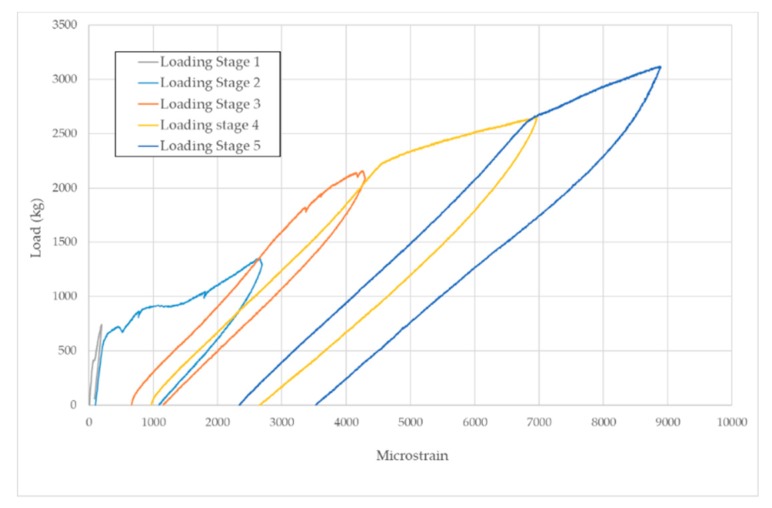
Loading–unloading curves for specimen A.

**Figure 6:**
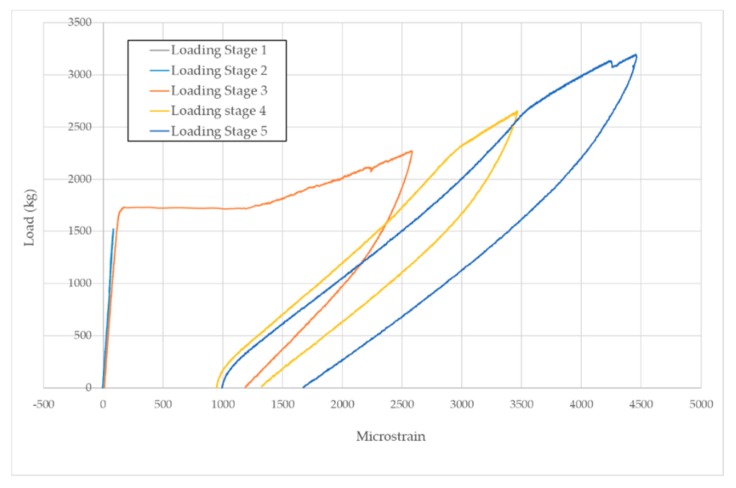
Loading–unloading curves for specimen B.

Concerning the loading procedure, from the baseline stage (D0) five loading steps (D1 to D5) were applied on both specimens until reaching the failure. Figure 5 and Figure 6 show the loading-unloading curves for each loading step for specimens A and B, respectively. The microstrain shown in Figure 5 was captured with a strain sensor bonded to the FRP bar and located where the left load point applies. On the other hand, microstrain in Figure 6 corresponds to a sensor also bonded to the FRP bar but located close to the debonded area between sensors PZT8 and PZT9 (Figure 2). For the specimen A, first cracks appeared during the second loading stage. These cracks grew during the subsequent loading stages and, additionally, yielding initiated during the fourth loading stage. The process is similar for specimen B although yielding initiated during the third loading stage. In Figure 6 it is difficult to identify the first cracks during the second loading stage since the strain sensor is located far from the midspan, where the first cracks appeared. 

The electromechanical impedance at each sensor was measured initially and after each loading step by exciting a wide frequency band of 10–100 kHz using each one of the PZT transducers and acquiring the response signal with the same transducer. An HP 4192A analyzer from Agilent was used. To measure several transducers, the device was coupled with a 3499B multiplexor from Agilent as well. Five frequency sweeps were conducted for each sensor at each damage state, resulting in five impedance signals. Therefore, 35 measurements were taken by each PZT transducer, although the signals after step 6 were not very useful considering that the beam was already severely damaged. 

The selection of a suitable frequency range is very important and, in fact, it is a problem to be solved in the application of EMI technique. In this study, the frequency range used has been up to 100 kHz. Considering that lower frequency ranges result in a larger sensing area, we think that working with ranges below 100 kHz, a relatively large area will be covered which can be enough for our damage detection purposes. 

### 4.1. Impedance Measurements

**Figure 7:**
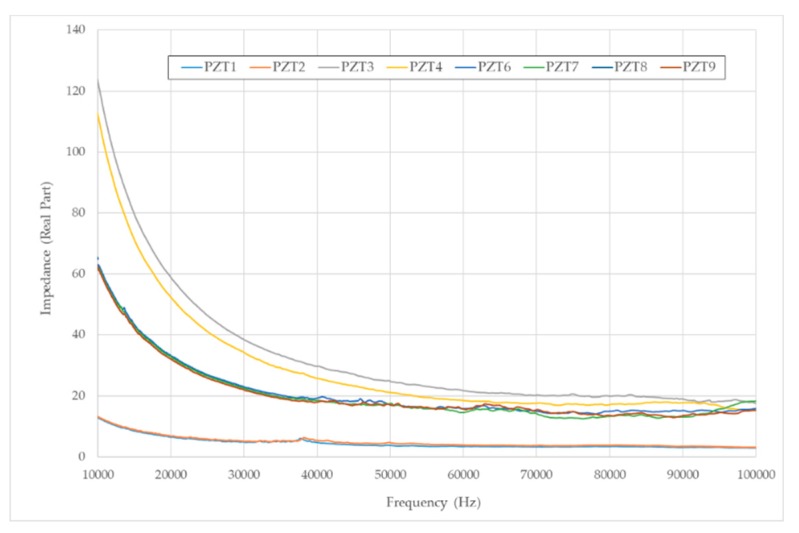
Impedance signals after the first loading stage—Specimen A.

Initially, the real part of the impedance measurements obtained with the eight sensors for specimen A after the first loading stage are shown in Figure 7. Clearly, the impedance signal shows a tendency to decrease with frequency for all sensors. However, the value of the impedance is not equal for all sensors. For the larger sensors, PZT1 and PZT2, the value of the impedance is significantly lower than for the rest of sensors. Moreover, the embedment of the sensors, PZT6 to PZT9, makes the impedance signals to decrease significantly as compared to sensors PZT3 and PZT4, which are exactly the same type but surface-mounted instead of embedded. A similar behavior was observed for the rest of damage states and for specimen B. Impedances show that, for damage assessment purposes, three groups of sensors should be considered, depending on the type and their integration into the structure. 

**Figure 8:**
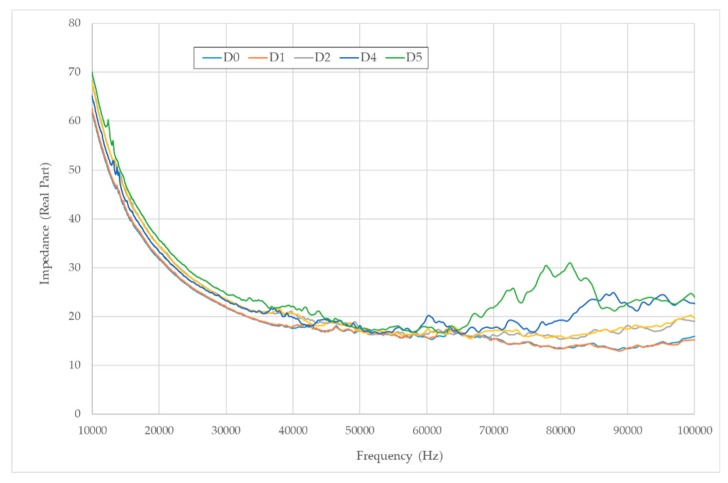
Change of the impedance signal for PZT9 along the different damage stages.

Additionally, Figure 8 shows how the impedance signal changes for a sensor from one damage state to another. Although, visually, this variation is low, it will serve us in the next sections to discriminate among different damage states by using the procedures proposed in Section 3.

Imaginary part of the impedance should mainly capture the changes due to temperature. Although not shown here by simplicity, negligible variations were observed between imaginary impedance signatures corresponding to two consecutive loading stages. During the tests the environmental conditions of the lab were kept constant since studying the effect of environmental variability on the system is outside the scope of this paper, and will be presented in future works. In this sense, variations in the real part of the impedance should be mainly due to mechanical damage of the specimens and will be used in the further clustering analysis. 

### 4.2. Damage Identification—Beam A

#### 4.2.1. Hierarchical clustering: Discussion

Once an initial clustering has been performed, the main study aimed at identifying sensors and stages with abnormal responses with the purpose of predicting the possible failure of the specimen. In this first test beam, the failure was due to a tensile fracture of the FRP bar on the right side of the middle section. It occurred suddenly, which makes difficult the identification by PZT sensors. However, all sensors have been inspected to check if they provide relevant information of severe damage in the last load steps. 

**Figure 9:**
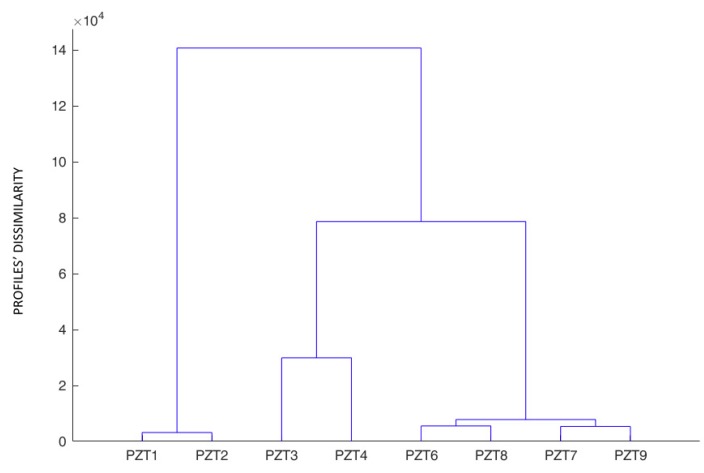
Hierarchical tree for specimen A—baseline stage.

Initially, the dendrogram is constructed for all sensors and the baseline stage (Figure 9). It shows clearly the grouping is dependent on the type of sensor and its location. Sensors of the same type, PZT1 and PZT2, are grouped while the remaining sensors belonging to the same type are grouped depending on if they were bonded on the external face of the beam or directly on the internal FRP bar.

**Figure 10:**
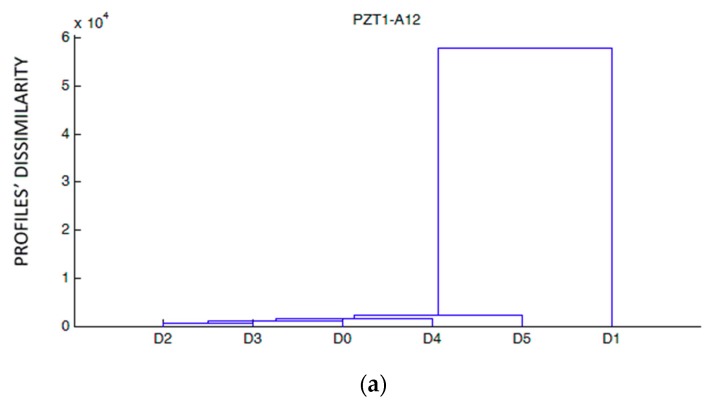

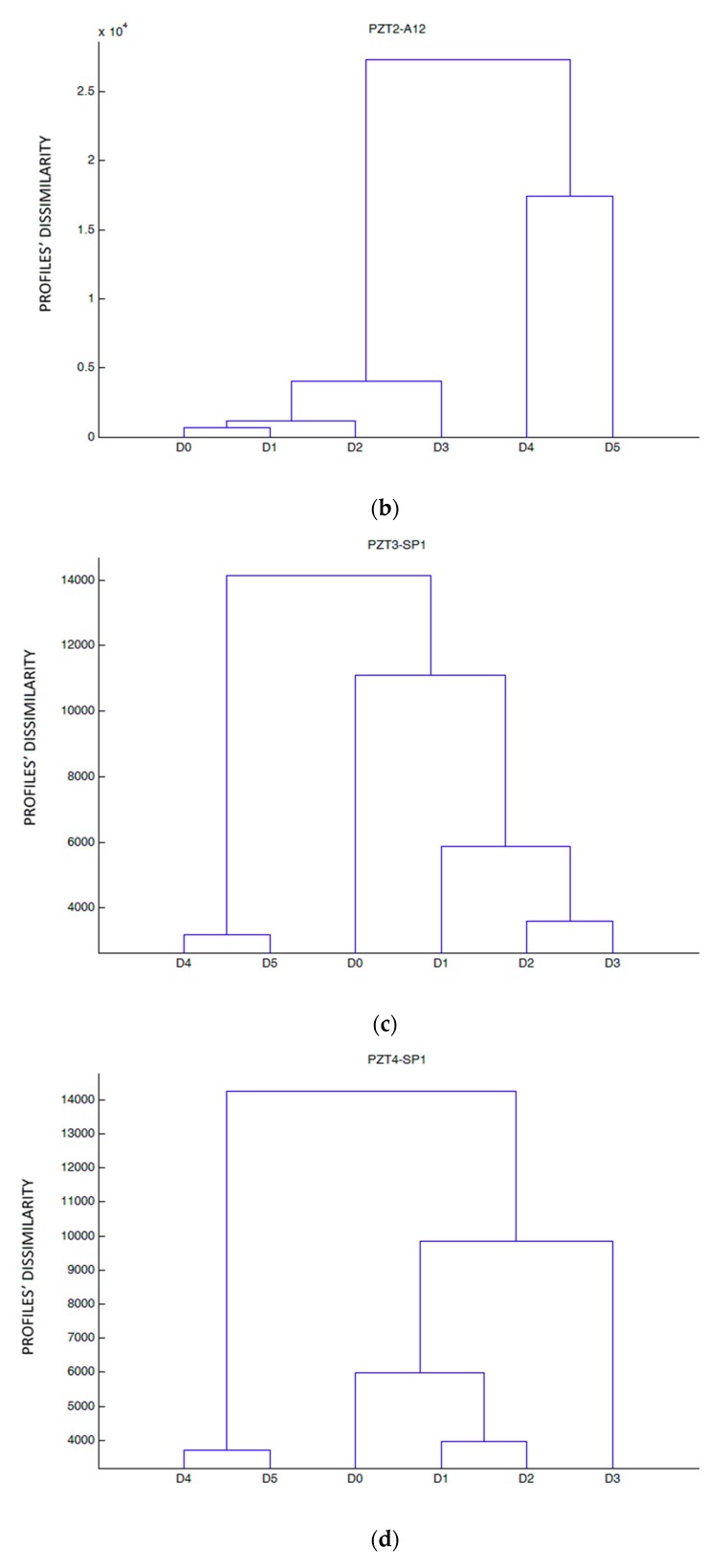

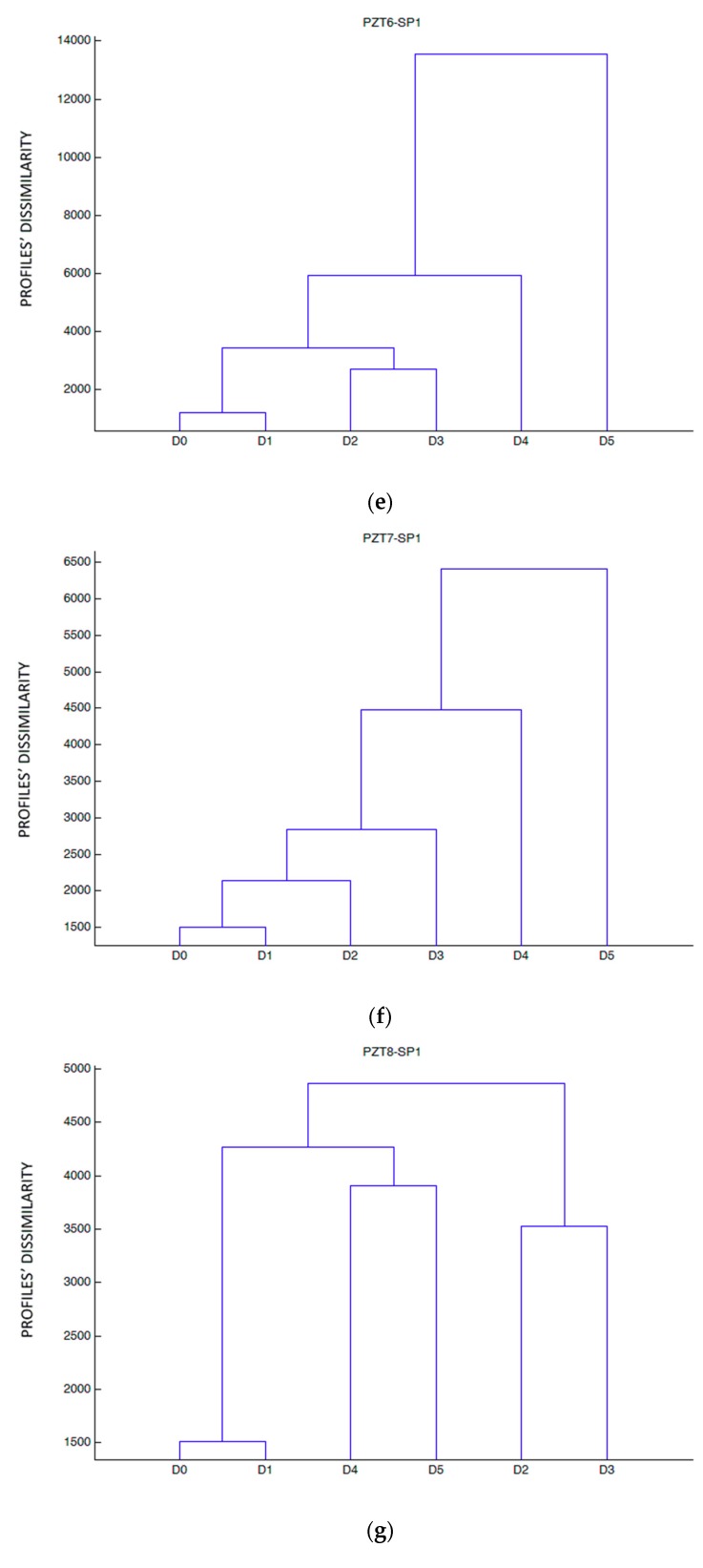

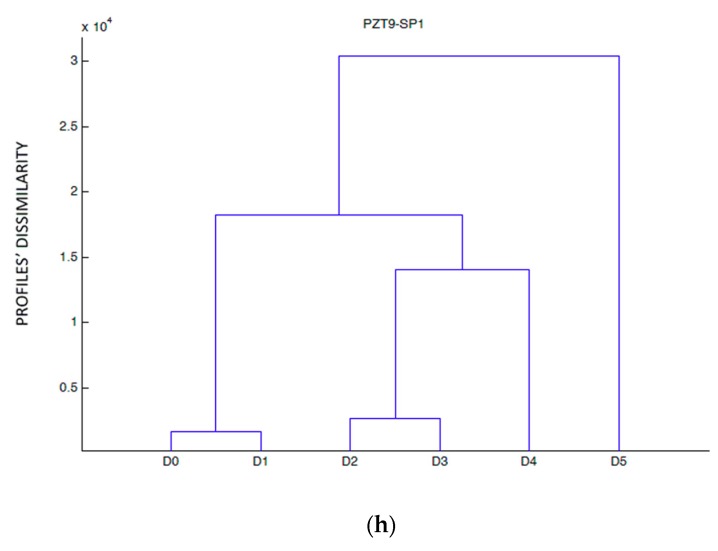
Hierarchical trees for specimen A. (**a**) Sensor PZT1, (**b**) Sensor PZT2, (**c**) Sensor PZT3, (**d**) Sensor PZT4, (**e**) Sensor PZT6, (**f**) Sensor PZT7, (**g**) Sensor PZT8, (**h**) Sensor PZT9.

Figure 10 shows the dendrograms constructed for each sensor and damage stage by using the raw impedance data. From the observation of the results, it is clear that, in general, internal sensors, PZT6, PZT7, PZT8, and PZT9, show a different performance of the external sensors. They differentiate clearly damage stages D4 and D5 from the other stages. Sensors PZT6 and PZT7, both internally located on the right of the middle section, show a progressive damage increment, higher in the last two loading stages. This information might indicate that the damage has reached more severely the internal areas of the beam around the FRP bar. For sensor PZT8, this effect is clear for the last two loading stages, where the reached levels of damage are similar. In sensor PZT9, almost just where damage occurred, a sudden growth of damage occurred in the last loading stage. This might serve as a warning for an imminent failure of the beam.

Sensors PZT3 and PZT4 can be gathered in the same group, since both of them differentiate between two clusters, one with the damage states 1–3 and another one with the damage states 4 and 5. However, they do not succeed in differentiating these two damage states, which can be due to the fact that the area around these sensors was subjected to a lower growth of cracks during the last loading steps. In addition, once the experiment concluded, no cracks were observed crossing the sensors. During the last two loading stages, growth of cracks occurred mainly internally as a previous step to the final failure.

With respect to the most external sensors on the concrete face, which are of the same type, PZT1 only detected the first minor cracks appearing during the first load stage. Two cracks surrounded this sensor during this step but no new crack appeared during the subsequent steps.

Sensor PZT2 shows a clear differentiation between damage states 4 and 5 and the rest of damage states. It should be noted that these two damage states are those in which the beam was the most severely damaged. Therefore, this sensor gives more useful information, since it is clearly able to distinguish the more severely damaged states.

#### 4.2.2. K-means clustering: Discussion

This methodology was applied for all sensors and loading stages using as feature vector the average third order moment (n = 3 in Equation (6)) from the five conducted frequency sweeps. Although not shown here, results for the second order SM were similar. The number of clusters was set to three in agreement with Figure 7 considering the different expected performance depending on the type of sensor and its location (internal or external). 

Table 1 shows the clustering results, i.e., the cluster number assigned to each sensor, from a 20-replication run along the five loading stages. The initial grouping agrees with conclusions of Figure 7, as expected. Then, some modifications occur whenever damage progresses. During the baseline and first loading stage, the grouping scheme shows a cluster with A12 type external sensors, a second cluster with SP1 type external sensors and a third cluster with all the SP1 internal sensors. From the second loading stage, sensor PZT9 moved to the same cluster grouping sensors PZT3 and PZT4. It should be remarked that, in this second stage, a crack crossed just below sensor PZT9 which clearly affected its performance. This clustering scheme did not change until the last loading scheme, for which sensor PZT9 moved to a one-member group; it is a symptom of anomaly associated to this sensor. Although an anomaly does not necessarily involve damage, since it can be due to any other phenomenon, if we combine this conclusion with those obtained from the hierarchical clustering, it is evident that a critical damage exists close to sensor PZT9, such as the experimental tests showed.

**Figure 11:**
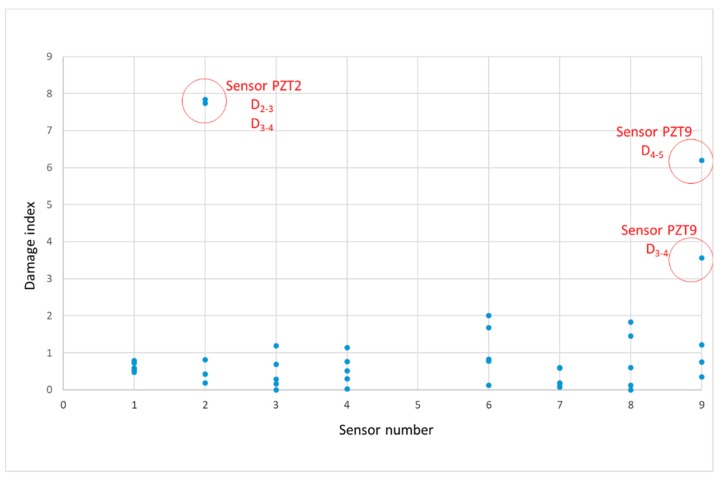
Damage index—beam A.

Figure 11 shows the damage index, computed according to Equation (5), of the entire observations (all sensors and loading stages). Damage indices for each sensor have been computed between consecutive loading stages. With the damage index we detect abnormal variations between two subsequent loading stages for the same sensor. Logically, if there is an important mechanical damage close to the sensor, this damage will be the responsible from the variation but any other anomaly, such as change in the environmental conditions or slight debonding of the sensor, might be the origin of the abnormal variation. From Figure 11, four outliers, two for sensor PZT2 and two for sensor PZT9, which can be interpreted as anomalies, were identified. It is important to remark that both sensors are on the right side of the beam, just close to the area where the beam failed. The anomalies associated to PZT2 occurred between stages 2 and 3 and stages 3 and 4 while those of sensor PZT9 correspond to the last two loading stages. It is in agreement with Figure 10b, where a strong growth of damage appeared in the fourth stage, and with Figure 10h and might be interpreted as a prelude of the subsequent failure. This conclusion agrees with Table 1 and the conclusions of the hierarchical clustering. 

### 4.3. Damage Identification—Beam B

#### 4.3.1. Hierarchical Clustering: Discussion

As commented previously, according to the analytical estimations, the predicted failure mode for this specimen should be the debonding of the FRP bar due to a loss of adherence. Furthermore, to favor this, some areas of the FRP bar were not adhered with epoxy to the concrete in order to simulate a defect at the concrete-FRP interface, such as shown in blue color in Figure 2. However, despite this, the failure mode of the beam was not the analytically predicted one, as the FRP showed a high bonding strength and a rupture of the composite material on the left of the middle section and close to the load point occurred.

The same test procedure than for beam A was performed with this beam. Impedance values associated to the baseline stage showed an anomalous behavior and, for this reason, were not used for further analysis.

**Figure 12:**
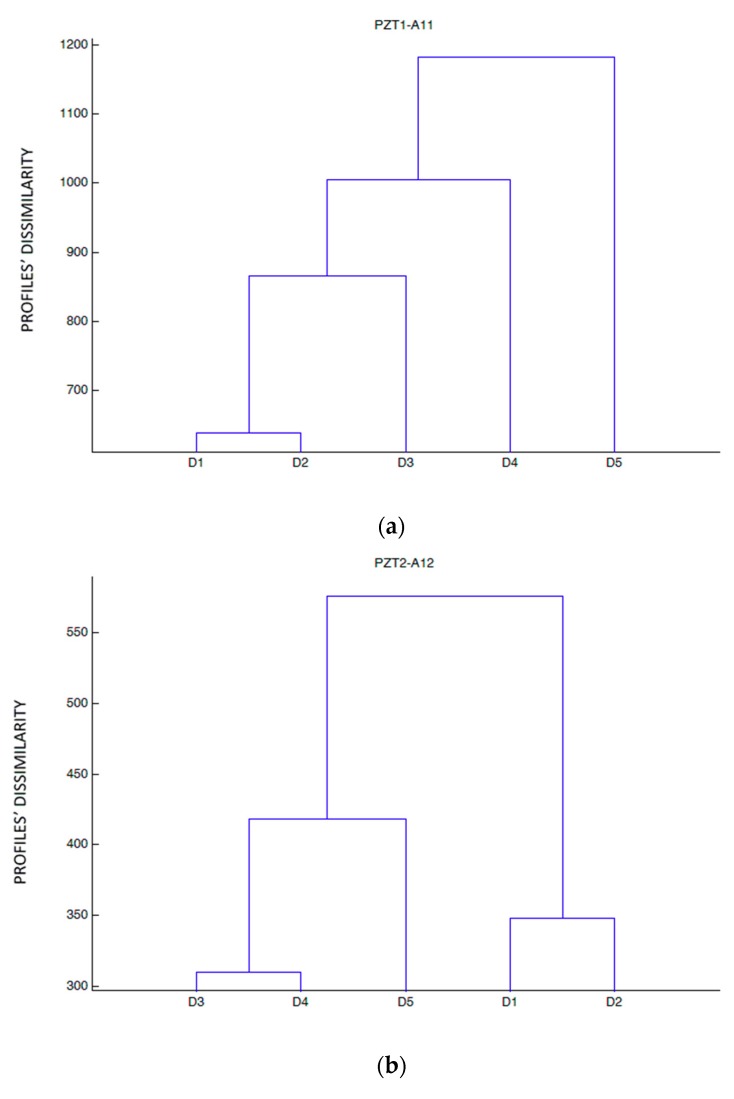

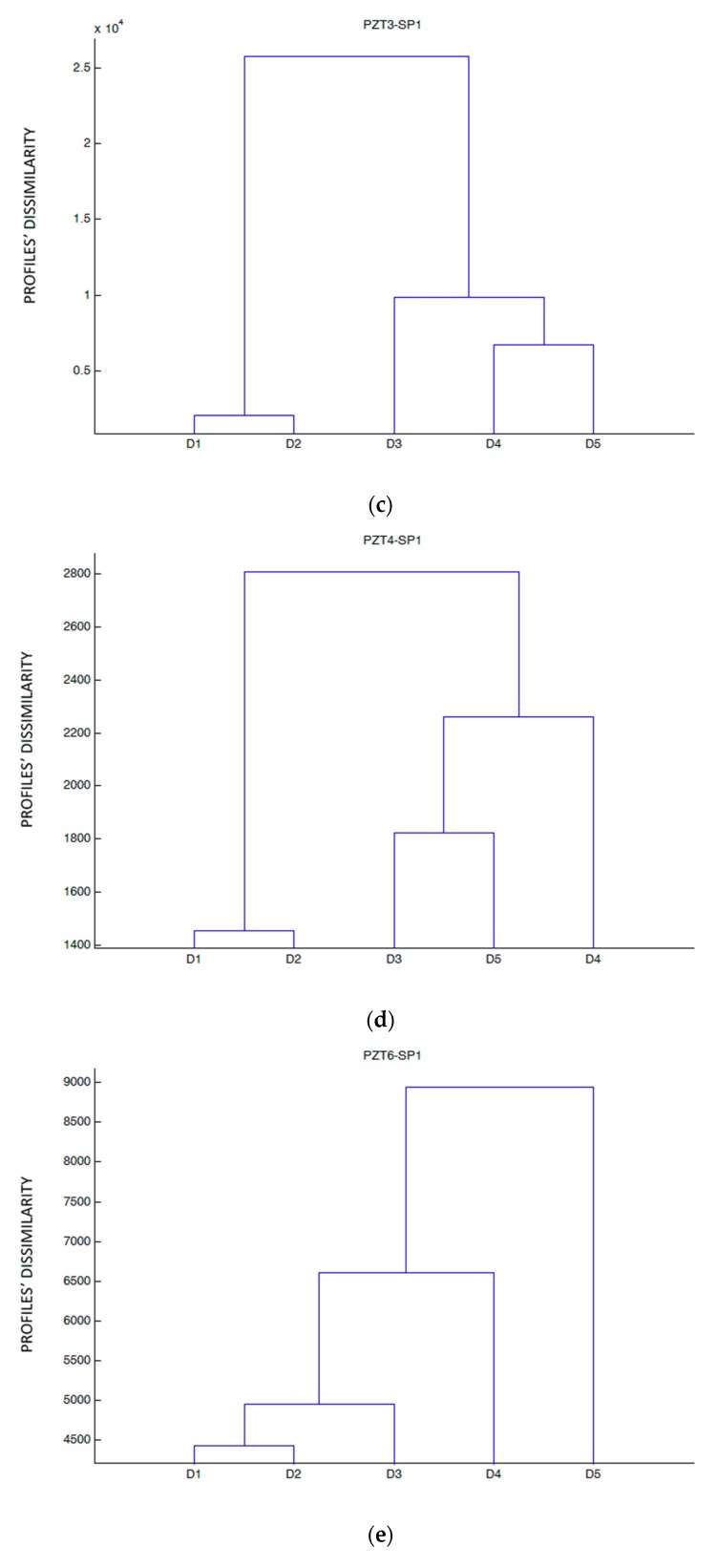

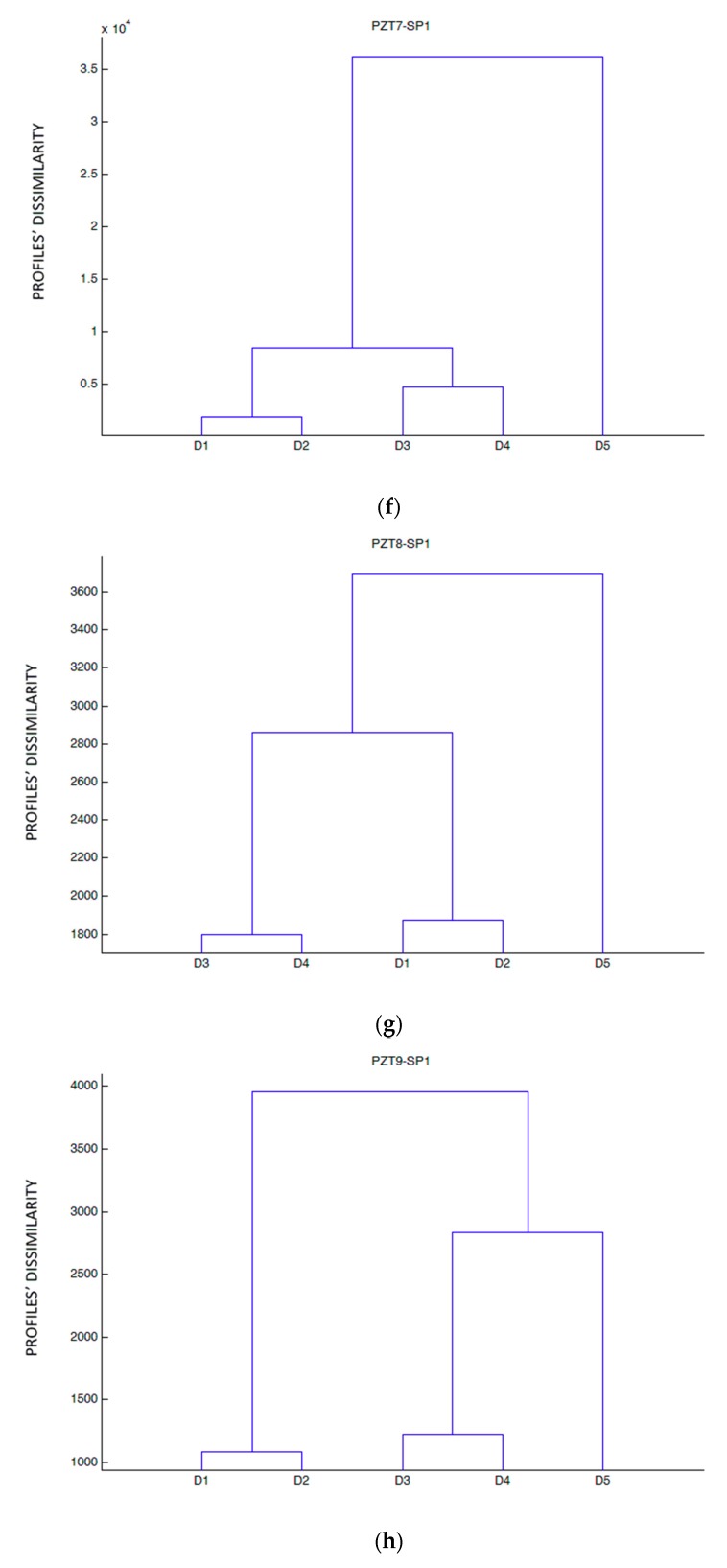
Hierarchical trees for specimen B. (**a**) Sensor PZT1, (**b**) Sensor PZT2, (**c**) Sensor PZT3, (**d**) Sensor PZT4, (**e**) Sensor PZT6, (**f**) Sensor PZT7, (**g**) Sensor PZT8, (**h**) Sensor PZT9.

Figure 12 shows the dendrograms constructed from the raw impedance data for all the sensors and all damage stages excluding baseline stage. The goal was to distinguish the five different damage stages (from 1 to 5).

From Figure 12, we are able to make a distinction among several groups of sensors depending on how they clustered the damage stages. In one group we include sensors PZT1 and PZT6, which were both able to show the progression of damage. This performance correlates well with the fact that they were the closest sensors to the beam midspan, and were, therefore, subjected to more severe damage. A second group would include sensors PZT2 and PZT9, the farthest to the midspan, which present small variations in the impedance signals and perform a similar clustering. As in the previous case, it is interesting to note that these sensors were located very close to each other. Another group would include external sensors PZT3 and PZT4, which present an analogous grouping of the damage states, although the variations for sensor PZT3 are more significant than for sensor PZT4. Finally, the last group would include internal sensors PZT7 and PZT8, which also cluster the damage stages similarly, but with more significant impedance variations for sensor PZT7, especially at the last damage stage where a severe damage growth is clearly identified.

In this specimen, unlike the previous specimen, the high and sudden severity of damage reached in the last loading stage for the internal sensors PZT6, PZT7, and PZT8 might be a symptom, even although failure by rupture of FRP occurred, of imminent loss of adherence at the FRP-concrete interface close to the artificially induced initial debonded areas. In fact, from the visual observation of the specimen, the initial debonded areas extended to regions close to these sensors because of the appearance and widening of internal cracks. Due to this, it was difficult to predict the failure mode which unexpectedly occurred. 

#### 4.3.2. K-Means Clustering

This methodology was applied in a similar way to specimen A but without considering the baseline results. Three clusters and 20 replications were set. 

Table 2 shows the clustering scheme along the different loading stages. The initial clustering is kept until the last loading stage where sensor PZT7 moved to a one-member group. This agrees with the conclusion of hierarchical clustering and with experimental observations since, after failure, a large crack on the right of sensor PZT7 and almost overlapping with the closest debonded area to the midspan was identified, which might be informative that the loss of adherence around of that area was near.

**Figure 13:**
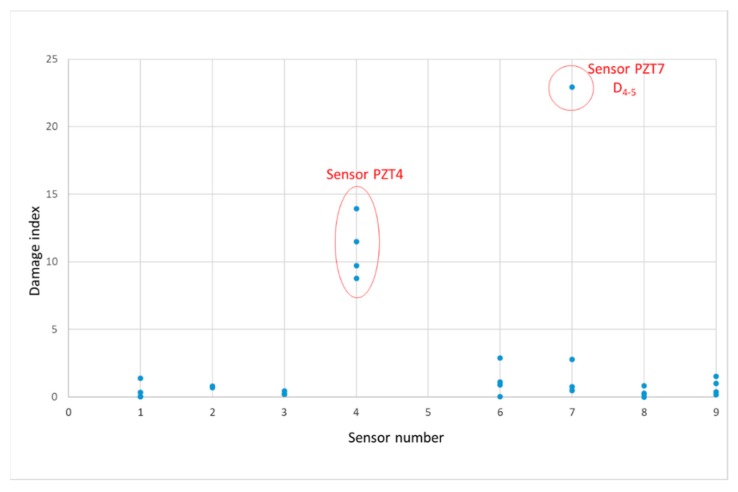
Damage index—beam B.

Figure 13 shows the damage index of the entire observations (all sensors and loading stages except baseline) computed between consecutive loading stages. From the plot, all the computations for sensor PZT4 can be considered as outliers and, additionally, an anomaly associated to sensor PZT7 is identified between the last two loading stages. This last outlier is consistent with the previous conclusions. However, the interpretation of the anomalies of PZT4 is difficult since although a crack appeared between sensors PZT3 and PZT4, we do not think it would justify the four outliers. Maybe, the main reason might be a measurement error in that sensor because of a partial peel-off of the sensor during the tests.

## 5. Conclusions

The early prediction of the failure of NSM-FRP strengthening systems is critical and remains an open issue. Typical failures initiate locally and, most of time, without previous warning. In this work, a high frequency range methodology, based on EMI, able to track in a fast, intuitive, and reliable way the evolution of the damages in concrete structures strengthened with NSM-FRP systems has been proposed.

EMI measurements captured with PZT sensors yield massive information covering a wide frequency range. A suitable processing of this information is key to implement a reliable detection system. In this work, a method combining unsupervised hierarchical clustering and k-means clustering has been developed. By applying these techniques, groups of complex measurements captured from several PZT sensors along different loading stages can be separated into clusters according to dissimilarity measures using two complementary methodologies. In one approach, clustering is performed for each sensor independently considering the different loading stages. In the second approach, grouping is made independently for each loading stage considering all the involved PZT sensors. Both approaches contain information from the entire frequency range by directly using raw impedance data or by extracting moments from the measured impedances. In this way, by combining conclusions of both alternatives, subtle differences between the normal signals and distorted ones and, thus, possible locally damaged areas could be identified. The performance evaluation of the proposed approach has been made successfully with two experimental tests on concrete beams strengthened with NSM-FRP. 

For future works, the damage detection procedure should be improved in order to consider varying and unknown conditions (operational and environmental) which are independent of the mechanical state of the structure but which might mask the effects due to damage. Variations due to operational and environmental changes should be discriminated from variations due to damage. This is, without doubt, an important challenge for the practical application of this proposed methodology in the SHM context.

## Figures and Tables

**Table 1:** K-means clustering result—beam A.

	Baseline	Stage 1	Stage 2	Stage 3	Stage 4	Stage 4
PZT1	1	1	1	1	1	1
PZT2	1	1	1	1	1	1
PZT3	2	2	2	2	2	2
PZT4	2	2	2	2	2	2
PZT6	3	3	3	1	3	2
PZT7	3	3	3	1	3	2
PZT8	3	3	3	1	3	2
PZT9	3	3	2	2	2	3

**Table 2:** K-means clustering results—beam B.

	Stage 1	Stage 2	Stage 3	Stage 4	Stage 4
PZT1	1	1	1	1	1
PZT2	1	1	1	1	1
PZT3	2	2	2	2	2
PZT4	2	2	2	2	2
PZT6	3	3	3	3	2
PZT7	3	3	3	3	3
PZT8	3	3	3	3	2
PZT9	3	3	3	3	2
